# Supplementary material for: A fault-intrusion-tolerant system and deadline-aware algorithm for scheduling scientific workflow in the cloud
Source: PeerJ Comput Sci. 2021 Nov 2;7:e747. doi: 10.7717/peerj-cs.747 (PMC8576554; doi:10.7717/peerj-cs.747)
Supplement: Supplemental Information 6 [file peerj-cs-07-747-s006.zip › Myproject1.1/docs/overview-summary.html]

Overview


JavaScript is disabled on your browser.


- Overview
- Package
- Class
- Use
- Tree
- Deprecated
- Index
- Help

- Prev
- Next

- Frames
- No Frames

- All Classes

Packages

| Package | Description |
| --- | --- |
|  |  |
| --- | --- |
| org.cloudbus.cloudsim |  |
| org.cloudbus.cloudsim.core |  |
| org.cloudbus.cloudsim.core.predicates |  |
| org.cloudbus.cloudsim.distributions |  |
| org.cloudbus.cloudsim.examples |  |
| org.cloudbus.cloudsim.examples.network |  |
| org.cloudbus.cloudsim.examples.network.datacenter |  |
| org.cloudbus.cloudsim.examples.power |  |
| org.cloudbus.cloudsim.examples.power.planetlab |  |
| org.cloudbus.cloudsim.examples.power.random |  |
| org.cloudbus.cloudsim.lists |  |
| org.cloudbus.cloudsim.network |  |
| org.cloudbus.cloudsim.network.datacenter |  |
| org.cloudbus.cloudsim.power |  |
| org.cloudbus.cloudsim.power.lists |  |
| org.cloudbus.cloudsim.power.models |  |
| org.cloudbus.cloudsim.provisioners |  |
| org.cloudbus.cloudsim.util |  |
| org.workflowsim |  |
| org.workflowsim.clustering |  |
| org.workflowsim.clustering.balancing |  |
| org.workflowsim.clustering.balancing.methods |  |
| org.workflowsim.clustering.balancing.metrics |  |
| org.workflowsim.examples |  |
| org.workflowsim.failure |  |
| org.workflowsim.planning |  |
| org.workflowsim.reclustering |  |
| org.workflowsim.scheduler |  |
| org.workflowsim.utils |  |

- Overview
- Package
- Class
- Use
- Tree
- Deprecated
- Index
- Help

- Prev
- Next

- Frames
- No Frames

- All Classes
